# Supplementary material for: Mutant IDH1 Differently Affects Redox State and Metabolism in Glial Cells of Normal and Tumor Origin
Source: Cancers (Basel). 2019 Dec 16;11(12):2028. doi: 10.3390/cancers11122028 (PMC6966450; doi:10.3390/cancers11122028)

cancers

MDPI

Supplementary Figures:

Mutant IDH1 differently affects redox-state and metabolism in glial cells of normal and tumor origin

Biedermann, Julia[1]; Preussler, Matthias[1]; Conde, Marina[2]; Peitzsch, Mirko[3]; Richter, Susan[3]; Wiedemuth, Ralf[2]; Abou-El-Ardat, Khalil[1]; Krüger, Alexander[1,4,5,6]; Meinhardt, Matthias[7]; Schackert Gabriele [2,5,6]; Leenders, William P.[8]; Herold-Mende, Christel[9]; Niclou, Simone P.[11]; Bjerkvig, Rolf[11,12]; Eisenhofer, Graeme[3,10]; Temme, Achim[2,5,6]; Seifert, Michael[5,13]; Kunz-Schughart, Leoni A.[4,5]; Schröck, Evelin[1,5,6]; Klink, Barbara[1,5,6,14*]

[1] Institute for Clinical Genetics, Faculty of Medicine Carl Gustav Carus, Technische Universität Dresden, Fetscherstraße 74, 01307 Dresden, Germany, Evelin.Schrock@tu-dresden.de
[2] Department of Neurosurgery, University Hospital Carl Gustav Carus, Technische Universität Dresden, 01307 Dresden, Germany, Achim.Temme@ukdd.de, Gabriele.Schackert@ukdd.de
[3] Institute of Clinical Chemistry and Laboratory Medicine, University Hospital Carl Gustav Carus, Technische Universität Dresden, 01307 Dresden, Germany, Susan.Richter@ukdd.de
[4] OncoRay - National Center for Radiation Research in Oncology, Faculty of Medicine and University Hospital Carl Gustav Carus, Technische Universität Dresden and Helmholtz-Zentrum Dresden-Rossendorf, 01307 Dresden, Germany, Leoni.Kunz-Schughart@oncoray.de
[5] National Center for Tumor Diseases (NCT), partner site Dresden, Germany
[6] German Cancer Consortium (DKTK), Dresden; German Cancer Research Center (DKFZ), Heidelberg, Germany
[7] Institute for Pathology, University Hospital Carl Gustav Carus, Technische Universität Dresden, 01307 Dresden, Germany, Matthias.Meinhardt@ukdd.de
[8] Department of Biochemistry, Radboud University Medical Center, Nijmegen, The Netherlands, William.Leenders@radboudumc.nl
[9] Experimental Neurosurgery, Department of Neurosurgery, University Hospital Heidelberg, Heidelberg, Germany, h.mende@med.uni-heidelberg.de
[10] Department of Medicine III, University Hospital Carl Gustav Carus, Technische Universität Dresden, 01307 Dresden, Germany, Graeme.Eisenhofer@ukdd.de
[11] Department of Oncology, NorLux Neuro-Oncology Laboratory, Luxembourg Institute of Health (LIH), Luxembourg, Luxembourg, simone.niclou@lih.lu
[12] Department of Biomedicine, University of Bergen, Bergen, Norway, Rolf.Bjerkvig@uib.no
[13] Institute for Medical Informatics and Biometry, Faculty of Medicine Carl Gustav Carus, Technische Universität Dresden, Germany, michael.seifert@tu-dresden.de
[14] National Center of Genetics (NCG), Laboratoire national de santé (LNS), Dudelange, Luxembourg, barbara.klink@lns.etat.lu

*Corresponding author.
E-mail: Barbara.klink@lns.etat.lu; Phone: +352 28100 418; Fax: - 441

Figure S1. Validation of Transduction Success:

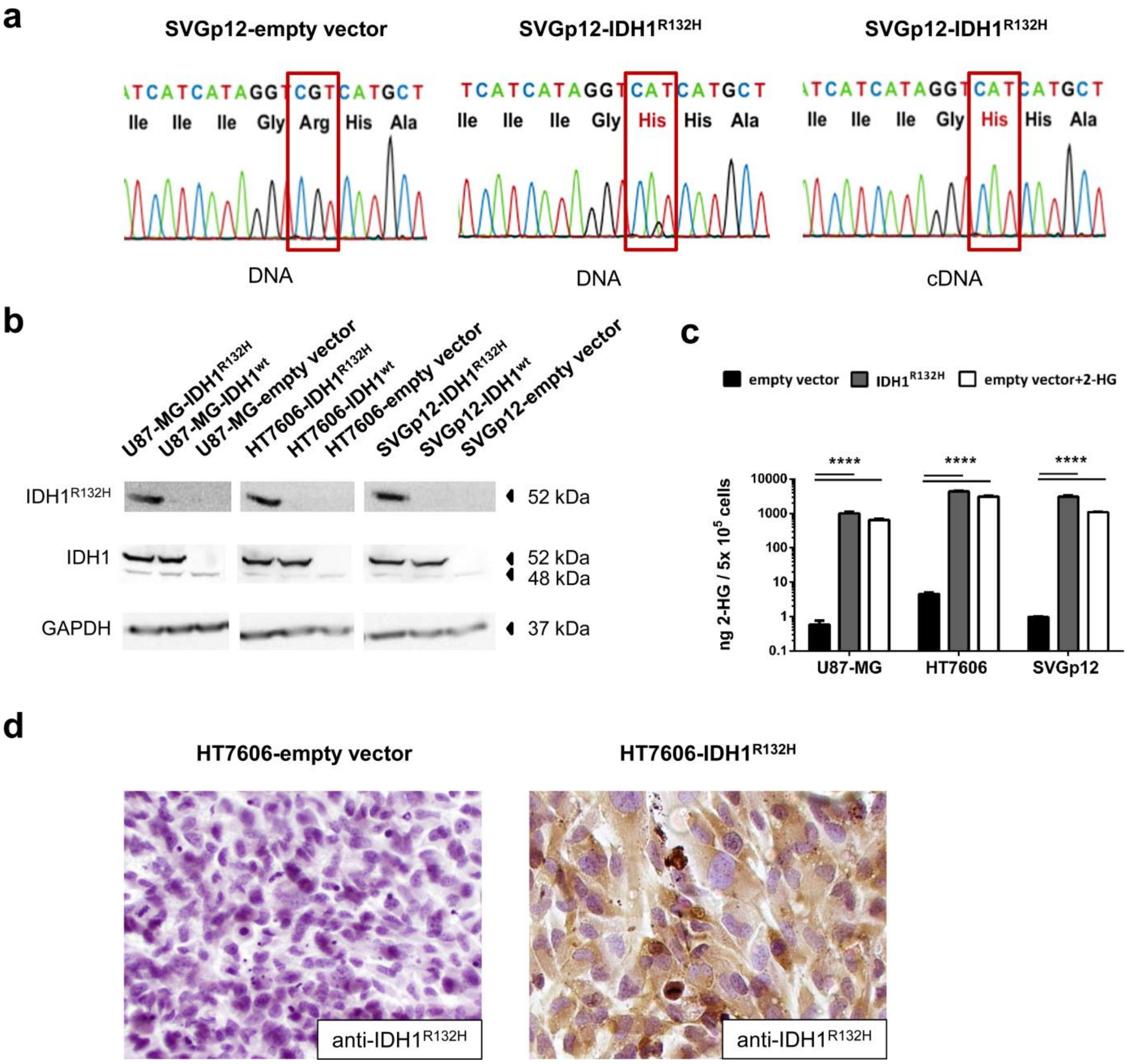

Figure S1. Validation of Transduction Success: We stably transduced the glioblastoma cell line U87-MG, a patient-derived glioblastoma cell line HT7606, and immortalized astrocytes SVGp12 with myc-tagged IDH1^{R132H}, IDH1^{wt}, or an empty vector as control and three independent transductions were performed per cell line for comparisons. a) Sanger sequencing confirmed wildtype IDH1 in empty vector control cells (left) and the IDH1 c.395G>A in IDH1^{R132H}-transduced cells on DNA (middle) and cDNA level (right). The example shows the result for one transduction of SVGp12. b) Western Blot analysis with the IDH1^{R132H} specific antibody confirmed IDH1^{R132H} expression (top). With an antibody detecting wildtype and mutant IDH1 (middle), a band at 52 kDa represents expression of myc-tagged IDH1^{R132H}/IDH1^{wt} and a smaller band at 48 kDa represents endogenous IDH1. Representative results for one transduction are given. c) Intra-cellular 2-HG levels were measured by LC-MS. Treatment of empty vector cells with 1 mM membrane permeable D-2-HG resulted in intra-cellular 2-HG levels comparable to that of IDH1^{R132H} transduced cells. d) IDH1^{R132H} protein expression was confirmed by immunocytochemical staining of cells grown on chambered cell culture slides using the IDH1^{R132H} antibody (1:20 dilution, H09, Dianova, Hamburg, Germany). Representative images of positively stained (brown) HT7606-IDH1^{R132H} (right) and negative HT7606-empty vector cells (left) are documented.

Figure S2. Comparison of absolute NADPt and NADPH levels:

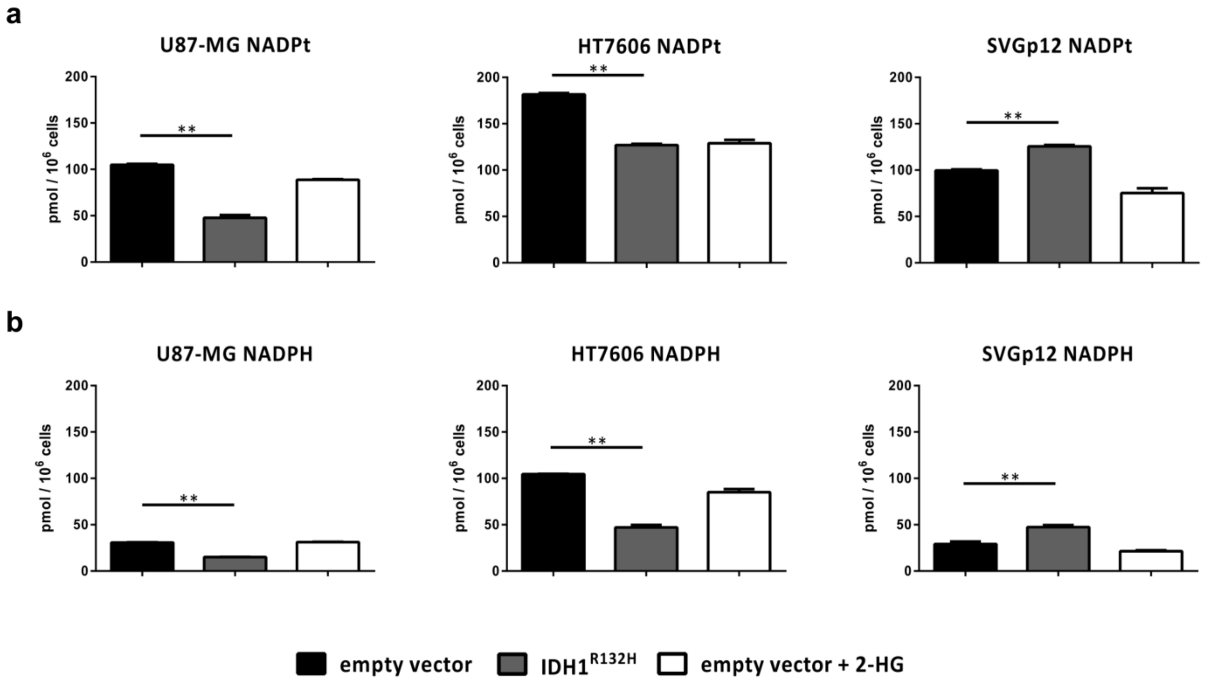

Figure S2. Comparison of absolute NADPt and NADPH levels in our cell models: NADPt (a) and NADPH (b) were measured in cell lysates from stably transduced cell lines as well as cells treated with D-2-HG only using the NADP+/NADPH Quantification Kit (MBL). Values were normalized to cell counts. Measurements were performed on cells from three independent transductions and in triplicates. Here, representative results from one experiment are shown. The primary glioblastoma cell line U87-MG and the immortalized astrocytes SVGp12 displayed similar intra-cellular levels for NADPt and NADPH, while HT7606 had higher levels. Transduction with IDH1R132H led to a significant drop in NADPt and NADPH in glioblastoma cells, but not in normal astrocytes SVGp12, which even showed elevated NADPt and NADPH levels. In contrast, treatment with D-2-HG alone did not change NADPH or NADPt levels. ** $p < 0.005$, using one-way analysis of variance (ANOVA) followed by Dunnett's post-hoc t-test

Figure S3. RNA expression of NAD-synthesizing enzymes:

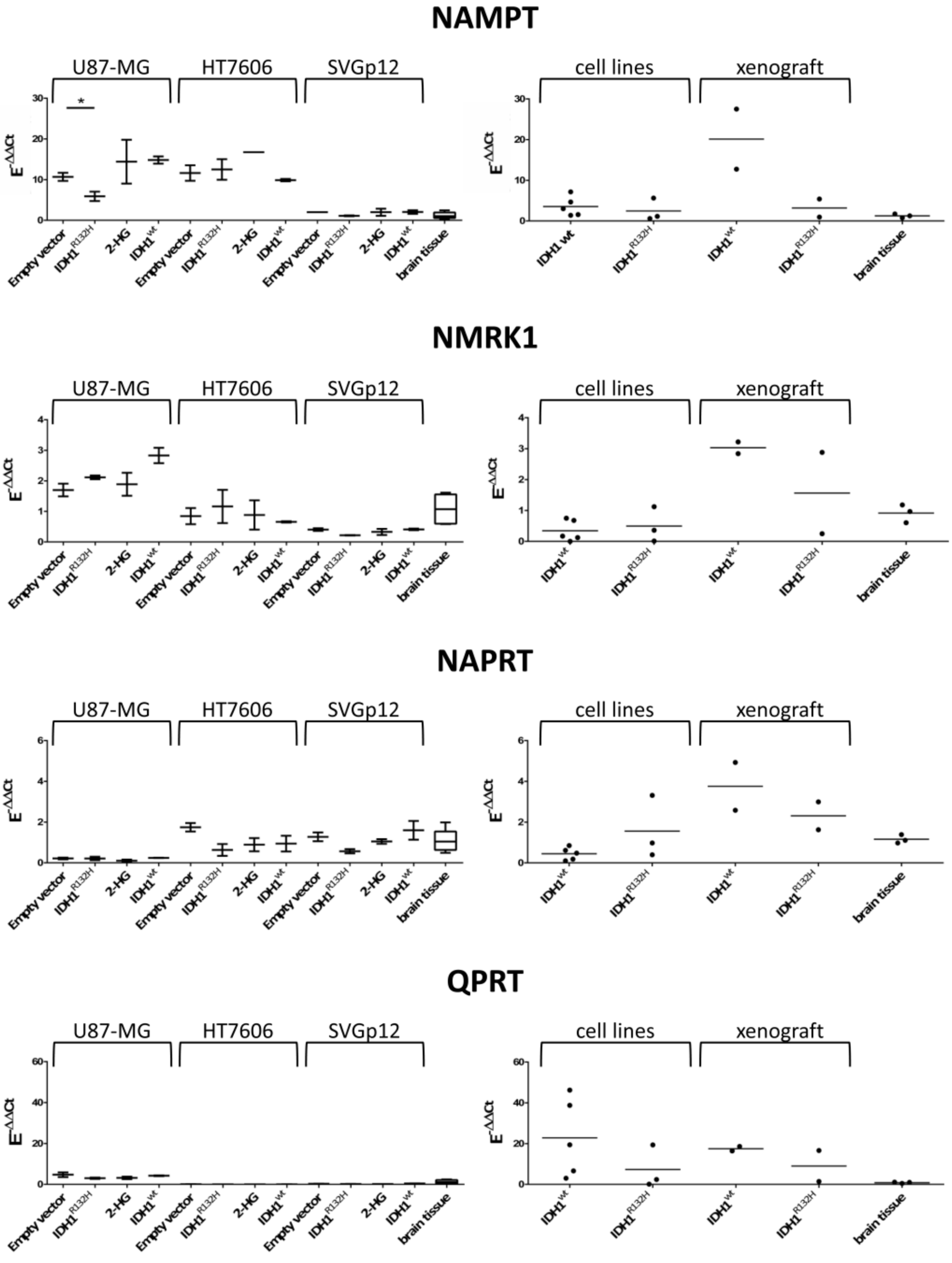

Figure S3. RNA expression of NAD-synthesizing enzymes in our cell models and in patient-derived glioma cell lines and xenografts: RNA expression levels of the rate-limiting enzymes in the four NAD-synthesis pathways: nicotin-amide phosphoribosyltransferase (NAMPT), nicotinic acid phosphoribosyltransferase (NAPRT), nicotinamide riboside kinase 1 (NMRK1), and quinolinic acid phosphoribosyltransferase (QPRT) were measured using reverse transcription quantitative PCR (RT-qPCR). Gene expression was normalized to the expression of reference genes *GAPDH* and *ARF1* ($E^{-\Delta CT}$) and thereafter to the expression in three commercially available RNAs from normal brain control tissues ($E^{-\Delta\Delta CT}$). Left: Results represented as median and SD are shown for our three cell line models (glioblastoma cell lines U87-MG, HT7606, and immortalized astrocytes SVGp12) that were stably transduced with empty vector, $IDH1^{R123H}$, and $IDH1^{wt}$, or treated with D-2-HG, respectively. Right: RNA expression levels in patient derived glioma cells with (n = 3) or without (n = 5) IDH mutation as well as two tissue samples each from a patient-derived glioma xenograft with and without IDH-mutation are

documented as median (bar) and dots representing individual samples. * $p < 0.05$, ** $p < 0.005$, using one-way analysis of variance (ANOVA) followed by Dunnett's post-hoc t-test

Figure S4. Protein expression of NAMPT:

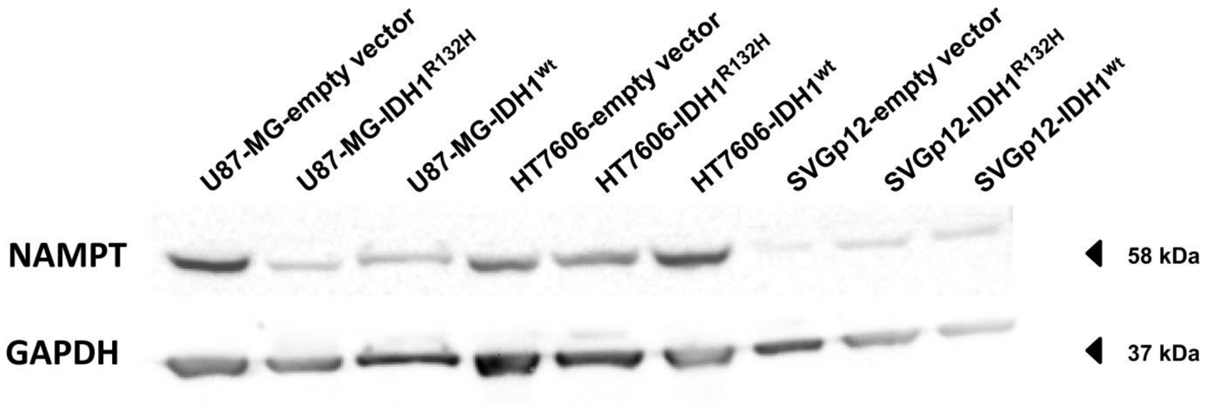

Figure S4. Protein expression of NAMPT in our cell models: Protein expression of the NAD-synthesis enzyme nicotinamide phosphoribosyltransferase (NAMPT) was analyzed by Western Blot and a representative blot is shown. The expression of NAMPT in control cells transduced with empty vector was lower for the normal astrocytes SVGp12 compared the glioblastoma cells U87-MG and HT7606. The glioblastoma cells had a much higher proliferation rate than the normal astrocytes *in vitro*, and therefor likely have a higher demand and turnover of NAD, which might explain the elevated NAMPT expression. Transduction with mutant IDH1 affected NAMPT expression differently in our cell models. While expression of NAMPT was induced in normal astrocytes upon IDH1^{R132H}-transduction, it was reduced in U87-MG and did not change in HT7606.

Figure S5. Protein expression of NAMPT:

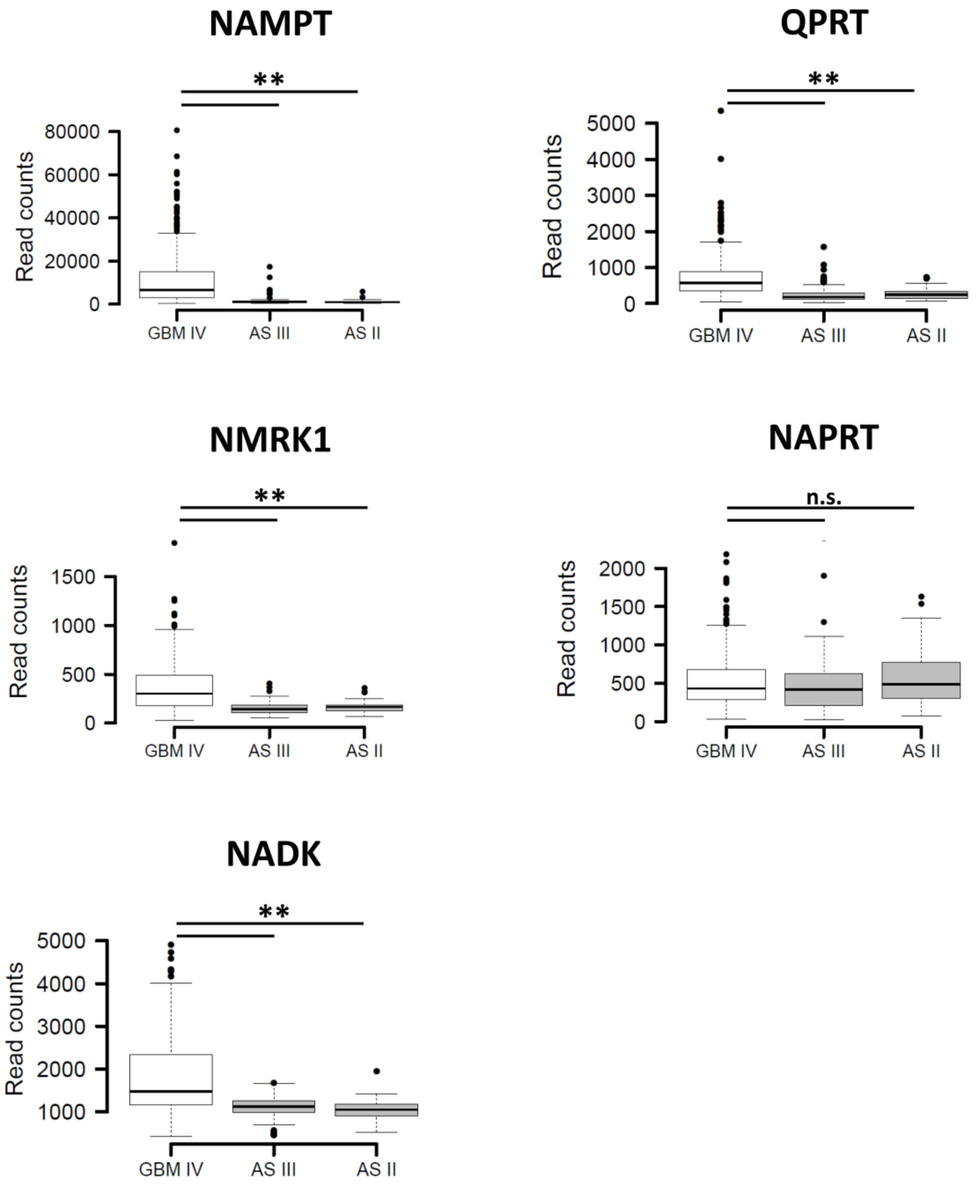

Figure S5. Expression of NAD-synthesis enzymes and NAD-Kinase in IDH-mutant and IDH-wildtype glioma tissues from patients: Gene expression analysis of key enzymes involved in NAD-synthesis was done using publicly available data from The Cancer Genome Atlas (TCGA) database for patient glioma samples (54 astrocytomas WHO grade II and 83 astrocytomas WHO grade III with *IDH1*/*IDH2* mutation, and 348 *IDH*-wildtype primary glioblastomas WHO grade IV). The gene-specific expression levels of glioblastomas against those in *IDH*-mutant astrocytomas were compared using Wilcoxon rank sum tests (** $p < 0.01$). The expression of the NAD synthesis enzymes *NAMPT*, *NMRK1*, and *QPRT* and the NADPH salvage pathway enzyme *NADK* was significantly elevated in *IDH*-wildtype glioblastomas compared to *IDH*-mutant gliomas, suggesting an increased demand and turnover of NAD in these highly malignant and proliferating tumors. No difference in expression levels were observed for *NAPRT*, likely because expression of *NAPRT* is restricted to normal residual cells within the tumor tissue. Abbreviations: NADK = NAD-Kinase, NAMPT = nicotinamide phosphoribosyltransferase, NAPRT = nicotinic acid phosphoribosyltransferase, NMRK1 = nicotinamide riboside kinase 1, QPRT = quinolinic acid phosphoribosyltransferase

Figure S6. Western Blots:

a) Western Blots Figure S1b

Density ratios were calculated with GAPDH density as protein reference and U87-MG-empty vector as sample reference for every blot.

IDH1^{R132H} 52 kDa (Figure S1b)

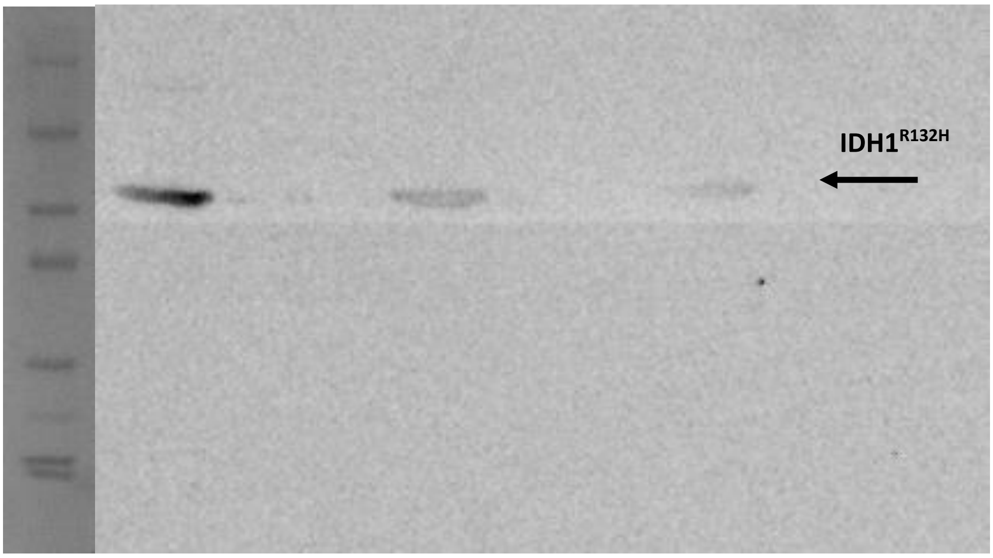

Sample	U87-MG-IDH1 R132H	U87-MG-IDH1 wt	U87-MG-empty vector	HT7606-IDH1 R132H	HT7606-IDH1 wt	HT7606-empty vector	SVGp12-IDH1 R132H	SVGp12-IDH1 wt	SVGp12-empty vector
Density Ratio	1.0	0	0	0.5	0	0	0.3	0	0

Repetition HT7606 and SVGp12 IDH1^{R132H} 52 kDa (Figure S1b)

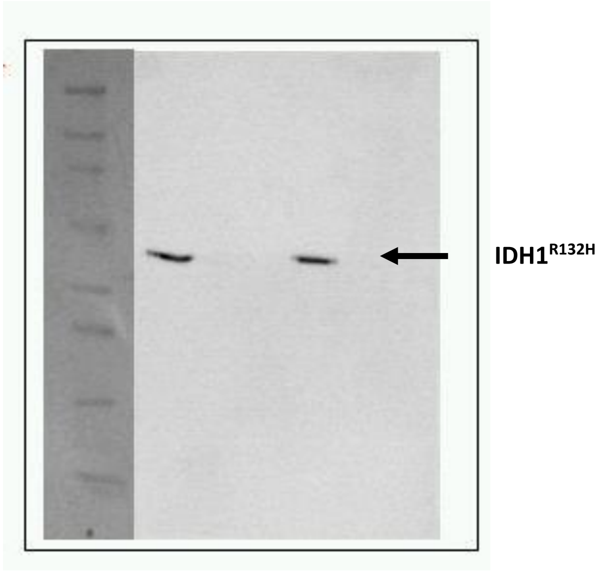

Sample	HT7606-IDH1 R132H	HT7606-IDH1 wt	HT7606-empty vector	SVGp12-IDH1 R132H	SVGp12-IDH1 wt	SVGp12-empty vector
Density Ratio	1.0	0	0	0.9	0	0

IDH1 52 kDa (Figure S1b)

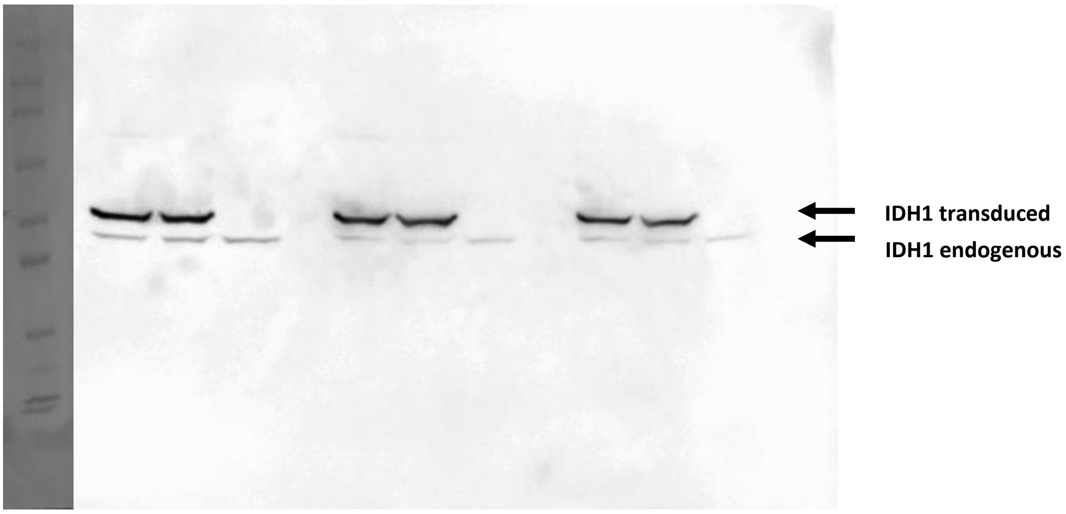

Sample	U87-MG-IDH1 R132H	U87-MG-IDH1 wt	U87-MG-empty vector	empty	HT7606-IDH1 R132H	HT7606-IDH1 wt	HT7606-empty vector	empty	SVGp12-IDH1 R132H	SVGp12-IDH1 wt	SVGp12-empty vector
Density Ratio	1.0	1.2	0		1.4	1	0		1	0.9	0

<u>GAPDH 37 kDa (Figure S1b)</u>

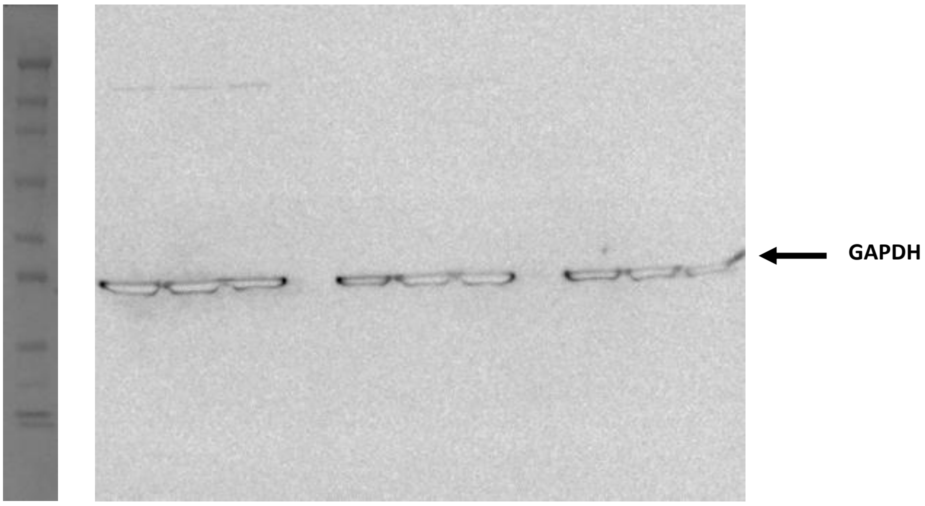

Sample	**U87-MG-IDH1 R132H**	**U87-MG-IDH1 wt**	**U87-MG-empty vector**	**empty**	**HT7606-IDH1 R132H**	**HT7606-IDH1 wt**	**HT7606-empty vector**	**empty**	**SVGp12-IDH1 R132H**	**SVGp12-IDH1 wt**	**SVGp12-empty vector**

b) Western Blots Figure 4b

Density ratios were calculated with GAPDH density as protein reference and U87-MG-empty vector as sample reference for every blot.

NAMPT 58 kDa (Figure 4b)

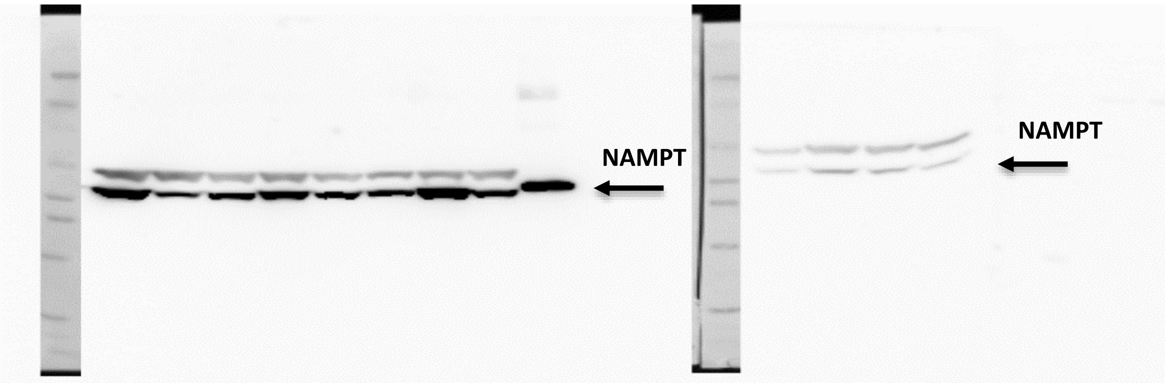

Sample	U87-MG-empty vector	U87-MG-IDH1 R132H	U87-MG-ev+ 2HG	U87-MG-IDH1 wt	HT7606-empty vector	HT7606-IDH1 R132H	HT7606-ev+ 2HG	HT7606-IDH1 wt	NAMPT recombinant protein	SVGp12-empty vector	SVGp12-IDH1 R132H	SVGp12-ev+ 2HG	SVGp12-IDH1 wt
Density Ratio	1.0	0.2	0.4	0.5	0.3	0.4	1.0	0.5	/	0.1	0.3	0.2	0.1

NMRK1 27 kDa (Figure 4b)

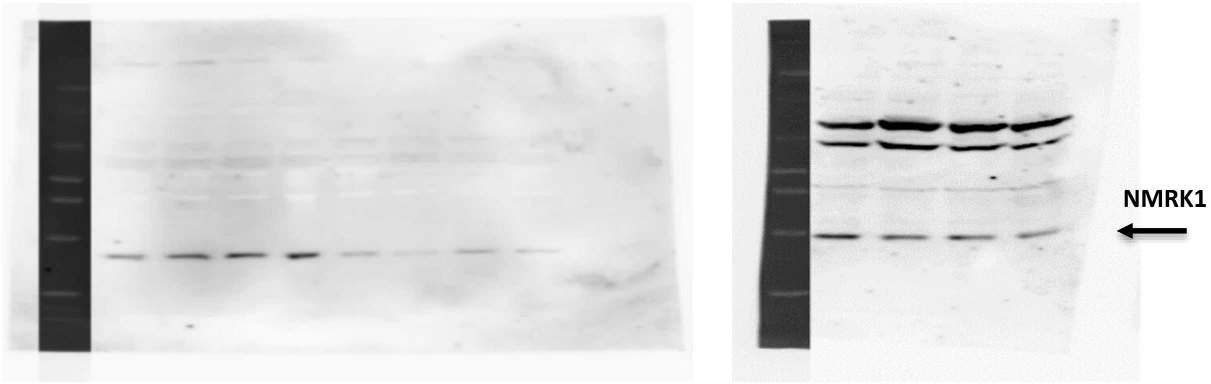

Sample	U87-MG-empty vector	U87-MG-IDH1 R132H	U87-MG-ev+ 2HG	U87-MG-IDH1 wt	HT7606-empty vector	HT7606-IDH1 R132H	HT7606-ev+ 2HG	HT7606-IDH1 wt	SVGp12-empty vector	SVGp12-IDH1 R132H	SVGp12-ev+ 2HG	SVGp12-IDH1 wt
Density Ratio	1.0	1.1	1.1	1.2	0.3	0.1	0.3	0.2	1.0	0.7	0.8	0.5

NAPRT 60 kDa (Figure 4b)

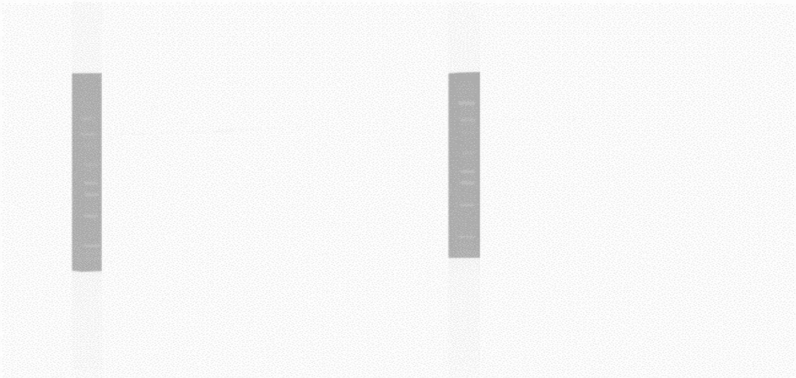

Sample	U87-MG-empty vector	U87-MG-IDH1 R132H	U87-MG-ev+ 2HG	U87-MG-IDH1 wt	HT7606-empty vector	HT7606-IDH1 R132H	HT7606-ev+ 2HG	HT7606-IDH1 wt	SVGp12-empty vector	SVGp12-IDH1 R132H	SVGp12-ev+ 2HG	SVGp12-IDH1 wt
Density Ratio	0	0	0	0	0	0	0	0	0	0	0	0

3-HAO 36 kDa (Figure 4b)

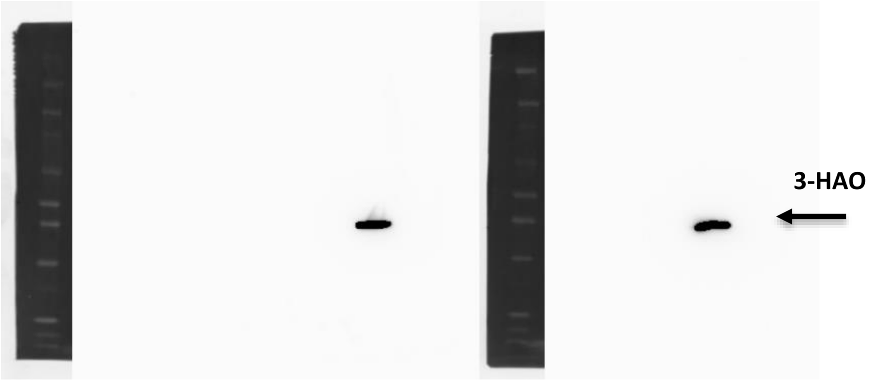

Sample	U87-MG-empty vector	U87-MG-IDH1 R132H	U87-MG-ev+ 2HG	U87-MG-IDH1 wt	HT7606-empty vector	HT7606-IDH1 R132H	HT7606-ev+ 2HG	HT7606-IDH1 wt	3-HAO r. protein	SVGp12-empty vector	SVGp12-IDH1 R132H	SVGp12-ev+ 2HG	SVGp12-IDH1 wt	3-HAO r. protein
Density Ratio	0.0	0.0	0.0	0.0	0.0	0.0	0.0	0.0	1.0	0.0	0.0	0.0	0.0	1.0

QPRT 34 kDa (Figure 4b)

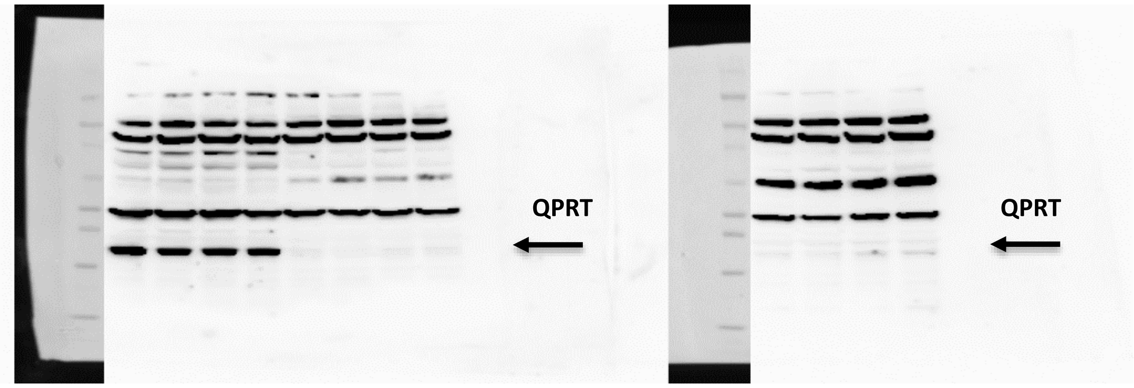

Sample	U87-MG-empty vector	U87-MG-IDH1 R132H	U87-MG-ev+ 2HG	U87-MG-IDH1 wt	HT7606-empty vector	HT7606-IDH1 R132H	HT7606-ev+ 2HG	HT7606-IDH1 wt	SVGp12-empty vector	SVGp12-IDH1 R132H	SVGp12-ev+ 2HG	SVGp12-IDH1 wt
Density Ratio	1.0	0.7	0.7	0.5	0.0	0.0	0.0	0.0	0.0	0.0	0.0	0.0

GAPDH 37 kDa (Figure 4b)

The reference GAPDH was detected after QPRT. The first four lanes and the last are QPRT protein detection bands. The second lane from the bottom is the GAPDH protein detection lane.

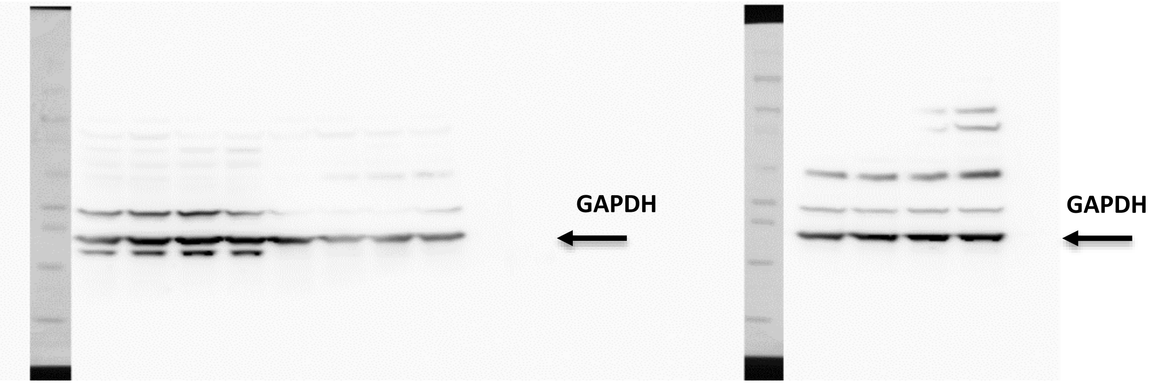

Western Blots Figure 4c

Density ratios are calculated with GAPDH density as protein reference and NCH551b as sample reference for every blot.

NAMPT 58 kDa (Figure 4c)

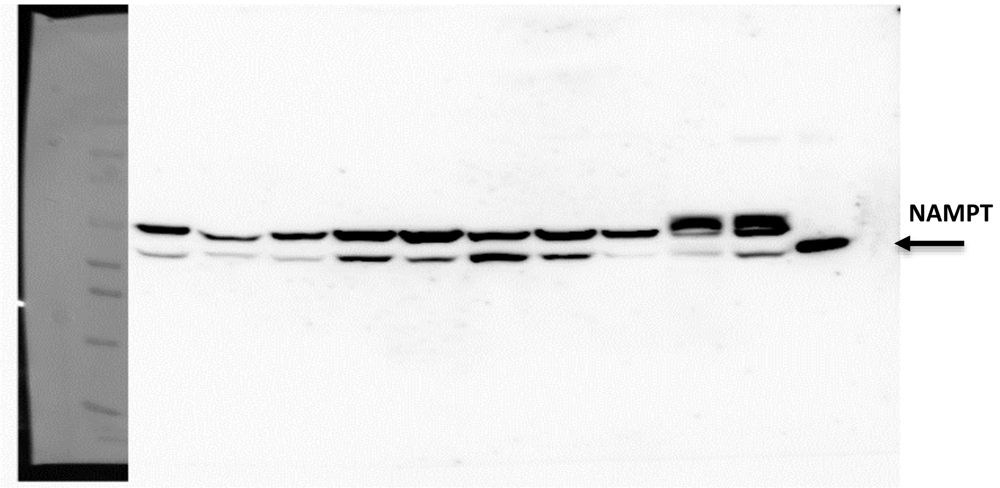

Sample	NCH551b	NCH612	NCH1681	NCH644	NCH421k	NCH601	NCH660h	NCH465	E478 CR500 '26'	P3 NC1293 '70'	NAMPT recombinant protein
Density Ratio	1.0	0.4	0.4	3.6	1.5	6.0	3.3	0.1	1.0	2.2	/

NMRK1 27 kDa (Figure 4c)

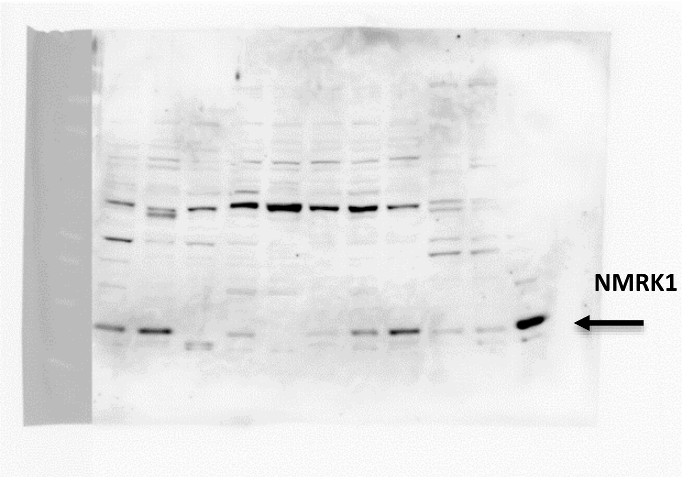

Sample	NCH551b	NCH612	NCH1681	NCH644	NCH421k	NCH601	NCH660h	NCH465	E478 CR500 '26'	P3 NC1293 '70'
Density Ratio	1.0	1.3	0.0	0.1	0.0	0.0	0.4	1.0	0.3	0.2

NAPRT 60 kDA (Figure 4c)

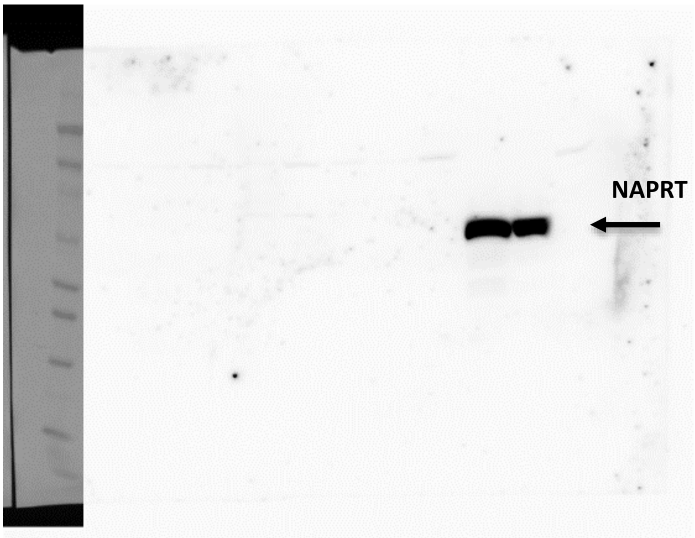

Sample	NCH551b	NCH612	NCH1681	NCH644	NCH421k	NCH601	NCH660h	NCH465	E478 CR500 '26'	P3 NC1293 '70'
Density Ratio	0.0	0.0	0.0	0.0	0.0	0.0	0.0	0.0	1.0	1.0

QPRT 34 kDa (Figure 4c)

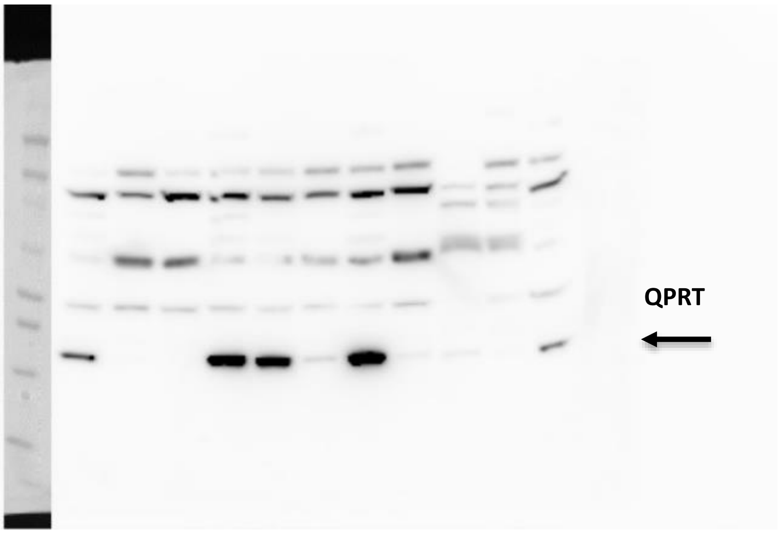

Sample	NCH551b	NCH612	NCH1681	NCH644	NCH421k	NCH601	NCH660h	NCH465	E478 CR500 '26'	P3 NC1293 '70'
Density Ratio	1.0	0.0	0.1	0.8	0.8	0.1	1.0	0.0	0.1	0.0

GAPDH 37 kDa (Figure 4c)

The reference GAPDH was detected after NAMPT. The first two lanes are NAMPT protein detection bands. The bottom lane is the GAPDH protein detection lane.

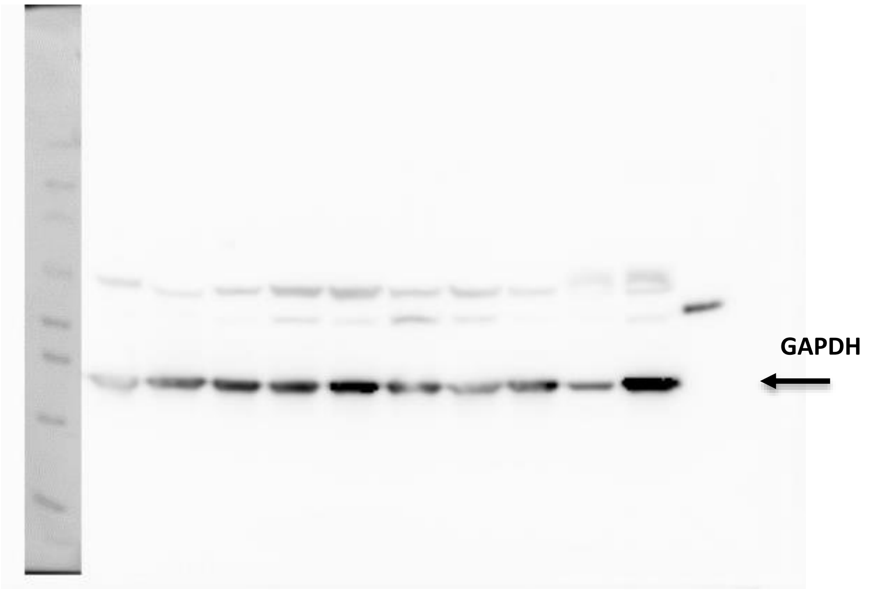

Supplement: Supplementary file 1 [file cancers-11-02028-s001.zip › cancers-625050-revised-supplementary Figures and western blot figures.pdf]
