# Supplementary material for: Mutant IDH1 Differently Affects Redox State and Metabolism in Glial Cells of Normal and Tumor Origin
Source: Cancers (Basel). 2019 Dec 16;11(12):2028. doi: 10.3390/cancers11122028 (PMC6966450; doi:10.3390/cancers11122028)
Supplement: Supplementary file 1 [file cancers-11-02028-s001.zip › cancers-625050-revised-supplementary tables.pdf]

cancers

MDPI

Mutant IDH1 differently affects redox-state and metabolism in glial cells of normal and tumor origin

Biedermann, Julia[1]; Preussler, Matthias[1]; Conde, Marina[2]; Peitzsch, Mirko[3]; Richter, Susan[3]; Wiedemuth, Ralf[2]; Abou-El-Ardat, Khalil[1]; Krüger, Alexander[1,4,5,6]; Meinhardt, Matthias[7]; Schackert Gabriele [2,5,6]; Leenders, William P.[8]; Herold-Mende, Christel[9]; Niclou, Simone P.[11]; Bjerkvig, Rolf[11,12]; Eisenhofer, Graeme[3,10]; Temme, Achim[2,5,6]; Seifert, Michael[5,13]; Kunz-Schughart, Leoni A.[4,5]; Schröck, Evelin[1,5,6]; Klink, Barbara[1,5,6,14*]

[1] Institute for Clinical Genetics, Faculty of Medicine Carl Gustav Carus, Technische Universität Dresden, Fetscherstraße 74, 01307 Dresden, Germany, Evelin.Schrock@tu-dresden.de
[2] Department of Neurosurgery, University Hospital Carl Gustav Carus, Technische Universität Dresden, 01307 Dresden, Germany, Achim.Temme@ukdd.de, Gabriele.Schackert@ukdd.de
[3] Institute of Clinical Chemistry and Laboratory Medicine, University Hospital Carl Gustav Carus, Technische Universität Dresden, 01307 Dresden, Germany, Susan.Richter@ukdd.de
[4] OncoRay - National Center for Radiation Research in Oncology, Faculty of Medicine and University Hospital Carl Gustav Carus, Technische Universität Dresden and Helmholtz-Zentrum Dresden-Rossendorf, 01307 Dresden, Germany, Leoni.Kunz-Schughart@oncoray.de
[5] National Center for Tumor Diseases (NCT), partner site Dresden, Germany
[6] German Cancer Consortium (DKTK), Dresden; German Cancer Research Center (DKFZ), Heidelberg, Germany
[7] Institute for Pathology, University Hospital Carl Gustav Carus, Technische Universität Dresden, 01307 Dresden, Germany, Matthias.Meinhardt@ukdd.de
[8] Department of Biochemistry, Radboud University Medical Center, Nijmegen, The Netherlands, William.Leenders@radboudumc.nl
[9] Experimental Neurosurgery, Department of Neurosurgery, University Hospital Heidelberg, Heidelberg, Germany, h.mende@med.uni-heidelberg.de
[10] Department of Medicine III, University Hospital Carl Gustav Carus, Technische Universität Dresden, 01307 Dresden, Germany, Graeme.Eisenhofer@ukdd.de
[11] Department of Oncology, NorLux Neuro-Oncology Laboratory, Luxembourg Institute of Health (LIH), Luxembourg, Luxembourg, simone.niclou@lih.lu
[12] Department of Biomedicine, University of Bergen, Bergen, Norway, Rolf.Bjerkvig@uib.no
[13] Institute for Medical Informatics and Biometry, Faculty of Medicine Carl Gustav Carus, Technische Universität Dresden, Germany, michael.seifert@tu-dresden.de
[14] National Center of Genetics (NCG), Laboratoire national de santé (LNS), Dudelange, Luxembourg, barbara.klink@lns.etat.lu

*Corresponding author. E-mail: Barbara.klink@lns.etat.lu; Phone: +352 28100 418; Fax: - 441

Supplementary Tables

Table S1. qPCR Primes for cDNA

primer name	sequence	product length
cNAMPT_Exon1_forward	GGCAGAAGCCGAGTTCAA	82 bp
cNAMPT_Exon2_reverse	GCTTGTGTTGGGTGGATATTG	
cNAPRT1_Exon11_forward	ACTGGTGCCAATGCCAAT	107 bp
cNAPRT1_Exon10_reverse	GGCTGGAGTCAGTCCTCATC	
cNmrk1_Exon8_forward	ATCAGCCTCCAGACTCTCCG	125 bp
cNmrk1_Exon9_reverse	CTCTTCAGATTTTGTTCCATCCA	
cQPRT_Exon2_forward	GAGGGCTGGTGATGGTGA	115 bp
cQPRT_Exon4_reverse	CTGCATTCCACTTCCACCTT	
cARF1_Exon2_forward	GACCACGATCCTCTACAAGC	111 bp
cARF1_Exon3_reverse	TCCCACACAGTGAAGCTGATG	
cGAPDH_Exon6_forward	ATGTTCGTCATGGGTGTGAA	89 bp
cGAPDH_Exon7_reverse	GGTGCTAAGCAGTTGGTGGT	

Table S2. Antibodies (Ab) applied for western blotting (WB).

Primary Ab	Clone	Host	Species reactivity*	Distributor	dilution/ working
IDH1 R132H	H09	Ms	H	Dianova	1:500
IDH1	2H9	Ms	H	Abcam	1:2000
QPRT	5D11	Ms	H	Sigma Aldrich	2 µg/ml
NAPRT1	n.d.	Ms	H	Sigma Aldrich	2 µg/ml
HAAO	n.d.	Ms	H	Abnova	2 µg/ml
NAMPT	P4D5AT	Ms	H	Enzo Life Science	1:2000
GAPDH	n.d.	Ms	H, B, Gt Ms, Rb, Rt Sw, F	Meridian Life Science	1:20.000
**Secondary Ab**					
Mouse IgG	n.d.	Gt	Ms	Santa Cruz Biotechnology	1:2000

* species reactivity according to distributor
n.d. - not defined, B – bovine, F-fish, Gt - goat, H- human, Ms - mouse, Rb - rabbit, Rt-Rat, Sw - swine
The primary antibodies were incubated at 4°C over night. The secondary antibody was incubated for 1 hour at room temperature.
